# Lipopolysaccharide Induces Immune Activation and SIV Replication in Rhesus Macaques of Chinese Origin

**DOI:** 10.1371/journal.pone.0098636

**Published:** 2014-06-11

**Authors:** Rong Bao, Ke Zhuang, Jinbiao Liu, Jianguo Wu, Jieliang Li, Xu Wang, Wen-Zhe Ho

**Affiliations:** 1 Animal Biosafety Level III Laboratory at the Center for Animal Experiment, Wuhan University, Wuhan, Hubei, P. R. China; 2 State Key Laboratory of Virology, Wuhan University, Wuhan, Hubei, P. R. China; 3 Department of Pathology and Laboratory Medicine, Temple University School of Medicine, Philadelphia, Pennsylvania, United States of America; University of Nebraska Medical Center, United States of America

## Abstract

**Background:**

Chronic immune activation is a hallmark of progressive HIV infection and a key determinant of immunodeficiency in HIV-infected individuals. Bacterial lipopolysaccharide (LPS) in the circulation has been implicated as a key factor in HIV infection-related systemic immune activation. We thus investigate the impact of LPS on systemic immune activation in simian immunodeficiency virus (SIV)-infected rhesus macaques of Chinese origin.

**Methods:**

The animals were inoculated intravenously with SIVmac239. The levels of plasma viral load and host inflammatory cytokines in PBMC were measured by real-time RT-PCR. CD4/CD8 ratio and systemic immune activation markers were examined by flow cytometric analysis of PBMCs. White blood cell and neutrophil counts and C Reactive Protein levels were determined using biochemistry analyzer. The plasma levels of LPS were determined by Tachypleus Amebocyte Lysate (TAL) test.

**Results:**

The animals inoculated with SIVmac239 became infected as evidenced by the increased plasma levels of SIV RNA and decreased CD4/CD8 ratio. LPS administration of SIV-infected animals induced a transient increase of plasma SIV RNA and immune activation, which was indicated by the elevated expression of the inflammatory cytokines and CD4+HLA-DR+ T cells in PBMCs.

**Conclusions:**

These data support the concept that LPS is a driving factor in systemic immune activation of HIV disease.

## Introduction

HIV infection is characterized by systemic immune activation, which is a major cause of progressive HIV disease. Microbial translocation has been suggested as a possible mechanism of systemic immune activation in individuals with chronic HIV infection. Microbial translocation is one of the common lesions of HIV-infected patients, as HIV infection damages mucosal barrier of the gastrointestinal (GI) tract, resulting in the intestinal bacteria and their products such as lipopolysaccharide (LPS) enter into the blood circulation. LPS in the circulation has been implicated as a key microbial product that contributes to HIV infection-related systemic immune activation [1]. LPS levels were significantly higher in individuals with chronic HIV infection than the control group [1]. More importantly, increased LPS was bioactive in vivo and positively associated with the immune activation [1]. In addition, plasma LPS-binding protein (LBP) levels were also increased in HIV-infected individuals and correlated positively with soluble CD14 (sCD14) levels [1]. Plasma LPS levels could be suppressed by antiretroviral therapy, but remained higher than those of uninfected control subjects [2]. Therefore, elevated LPS levels in blood are likely responsible for systemic immune activation in chronic HIV infection.

Infection of macaques with simian immunodeficiency viruses (SIV) is currently the best animal model to study HIV infection and AIDS pathogenesis [3]–[5]. Using SIV-infected Indian rhesus macaques, Brenchely et al. demonstrated that circulating LPS was significantly increased and that gut bacteria were the source of LPS, as plasma LPS levels were reduced following treatment with antibiotics [1]. In contrast, microbial translocation and chronic immune activation do not occur in SIV-infected natural host monkeys such as African green monkeys (AGMs) or sooty mangabeys (SMs) [6]–[8] but occur in non-natural host monkeys [6], [9]. In contrast to SIV-infected Indian rhesus macaques, both SIV-infected and uninfected sooty mangabeys have low levels of plasma LPS and no differences in sCD14 levels [1]. The absence or limited chronic immune activation in natural SIV infection highlights the importance of immune activation to disease progression in HIV/SIV infections [10], [11]. Although it has been shown that administration of LPS to chronic SIV-infected natural host AGMs could induce immune activation and significant increase in viral replication [9], it is unclear whether LPS plays a role in systemic immune activation in non-natural hosts of SIV infection such as rhesus macaques. In the present study, we investigated the impact of LPS administration on systemic immune activation and viral replication in SIV-infected rhesus macaques of Chinese origin.

## Materials and Methods

### Ethics Statement

All study protocols were approved by the Institutional Animal Care and Use Committee (IACUC) of the Wuhan University School of Medicine (Wuhan, China) in accordance with the regulations of the National Institute of Health “Guide for the Care and Use of Laboratory Animals” and all details of animal welfare and steps taken to ameliorate suffering were in accordance with the recommendations of the Weatherall report, “The use of nonhuman primates in research”. The rhesus monkeys used in this study were from the Sichuan Ping’an Non-Human Primates Breeding and Research Center, Sichuan Province, China (Table 1). The animals were housed in an air-conditioned room with an ambient temperature of 16–26°C, a relative humidity of 40–70% and a 12-hour light-dark cycle at the Animal Bio-Safety Level-III (ABSL-III) laboratory of the Wuhan University School of Medicine which were monitored in real time by a computer-based recording system. The animals were individually housed in stainless steel wire-bottomed cages with sufficient space (800 mm wide, 800 mm depth and 1600 mm height) and provided with a commercial monkey diet. In addition to normal pellet food, fresh fruit was provided twice daily, and water was freely available at all times. Animal health was monitored daily by the animal care staff and veterinary personnel. Additional enrichment and welfare were provided; for example, we routinely introduced rings, perches, forage boxes, puzzle-feeders into the home cage environment and played music and video in the room. We believed these environmental enrichment helped reduce any potential stress related to the experiment. Physiologic parameters of the animal, such as heart rate, body temperature and blood pressure etc were monitored at constant intervals and pain was evaluated by veterinarian. Procedures were carried out carefully to minimizing suffering and stopped to cease pain if necessary. No animal was sacrificed in this study. LPS injection or blood collection was performed under anesthesia with intramuscular injection of ketamine hydrochloride (10 mg/kg) plus intramuscular injection of atropine (0.04 mg/kg). All efforts were made to minimize suffering.

**Table 1:** Animals Used for the Study.

Animal No.	Code	Sex	Age (yr)	Weight (kg)	SIVmac	Inoculation Route	CD4 T Cell** (cells/ml blood)
WSP3	60228052	F	4.8	6.15	239	I.V.*	1604
WSP4	60423902	F	4.8	5.45	239	I.V.	543
WSP5	50621562	F	5.6	6.40	239	I.V.	941
WSL3	60409608	F	4.9	6.0	239	I.V.	880
WSL4	61025154	F	5.5	6.1	239	I.V.	1260
WSL5	60726502	F	5.2	6.0	239	I.V.	794

*I.V. = intravenous;

**analyzed at day 315 (45 weeks) post SIV inoculation.

### Experimental Animals

Six female CRMs were enrolled in this study. At the beginning of the study, the animals were screened and found negative for infections of simian retrovirus D, SIV, simian T leukemia virus type 1 (STLV-1), Herpes virus B, Strongyloides Stercoralis, Pneumonyssus Sinicola. Skin test (PPD) and X ray were also performed at entry for all study animals to exclude potential carriers of mycobacterial tuberculosis. At the time of SIV inoculation, the age range of animals was 4–6 years old and their weight range was 4.6–5.6 kg (Table 1). These CRMs infected with SIVmac239 showed no significant lymphadenopathy and after three months, all SIV-infected CRMs controlled viral replication. These animals were followed up for more than 2 years and then subject to experimental LPS administration by randomly dividing into two groups. One group animals (n = 3) were injected with LPS and the other group animals (n = 3) were injected with saline.

### SIV Challenge

SIVmac239 strain was obtained from the National Institutes of Health, AIDS Research & Reference Reagent Program (Contributor: Dr. Ronald Desrosiers). The tissue culture infective doses (TCID50) of SIVmac239 were titrated on TZM-bl cells. The animals were intravenously inoculated with 0.5 ml of SIVmac239 (10^3^ TCID50) as previously described [12]. Plasma samples were collected during the course of study (315 days, 45 weeks) with more frequent samplings in the first 8 weeks postinfection.

### LPS Administration

LPS from *E. coli* K12 strain was purchased from InvivoGen (San Diego, CA). At day 315 post SIV-infection, three infected animals in group one were intravenously injected with a single dose (50 µg/kg) of LPS. Another three infected monkeys received saline as the control. After LPS administration, blood samples were collected at 0.5 h, 2 h, 6 h and then daily for 7 days. Plasma LPS levels were determined by a commercial Tachypleus Amebocyte Lysate (TAL) test (Xiamen Chinese Horseshoe Crab Reagent Manufactory Co., Ltd., Xiamen, China) according to the manufacturer’s instruction.

### Plasma Viral Load

Blood was collected using ethylenediamine tetraacetic acid (EDTA) as the anticoagulant. Plasma was frozen at −70°C. The plasma levels of SIV RNA were measured by a quantitative real-time PCR as previously described [13]. The oligonucleotide primers used for SIV gag were listed in Table 2.

**Table 2:** Primer Pairs for Real-Time RT-PCR.

Primer	Orientation	Sequences (5′→3′)
SIV gag	Forward	GCAGAGGAGGAAATTACCCAGTAC
	Reverse	CAATTTTACCCAGGCATTTAATGTT
IL-6	Forward	TGGCTGAAAAAGATGGATGCT
	Reverse	TTGCTCCTCACTACTCTCAAACCT
IL-8	Forward	ACTGAGAGTGATTGAGAGTGGAC
	Reverse	AACCCTCTGCACCCATGGTTC
IFN-α	Forward	GCCTGAAGGACAGACATGACTTT
	Reverse	GGATGGTTTGAGCCTTTTGG
TNF-α	Forward	GGCTCAGGCAGTCAGATCATC
	Reverse	GCTTGAGGGTTTGCTACAACATG
GAPDH	Forward	GTCTGGAAAAACCTGCCAAG
	Reverse	ACCTGGTGCTCAGTGTAGCC

### Flow Cytometry

The CD4 counts and CD4/CD8 ratios in peripheral blood were determined by flow-cytometry, using fluorescence-labeled monoclonal antibodies from BD Biosciences (San Jose, CA, USA).

### Hemopathologic Blood Counts and Plasma C Reactive Protein (CRP)

Whole blood specimens were stained with Wright-Giemsa stain. The counts of white blood cell (WBC), neutrophils, and lymphocytes were determined by Biochemistry Analyzer (Hitachi 7080, Japan). CRP was determined by Biochemistry Analyzer with the reagents from Wako Pure Chemical Industries (Osaka, Japan), using animal plasma isolated from coagulated blood of the study animals.

### Real-Time RT-PCR

Peripheral blood mononuclear cells (PBMCs) were isolated from blood of the study animals using Ficoll-Paque PLUS method according to the manufacturer’s instruction. Total RNA was extracted from PBMCs using Tri-Reagent (Molecular Research Center, Cincinnati, OH) as previously described [14]. Total cellular RNA (1 µg) was subjected to reverse transcription using the reagents from Promega (Madison, WI). The real time RT-PCR for the quantification of mRNA levels of interleukin (IL)-6, IL-8, interferon (IFN)-α, tumor necrosis factor (TNF)-α was performed with the iQ SYBR Green Supermix (Bio-Rad Laboratories, Hercules, CA). The levels of glyceraldehyde-3-phosphate dehydrogenase (GAPDH) mRNA were used as an endogenous reference to normalize the quantities of cytokine mRNAs. The oligonucleotide primers used for the cytokines were listed in Table 2.

### Statistical Analysis

All real-time PCR and bioparameter analyses were performed at least in triplicate. All statistical assessments were two-sided, and *P*<0.05 was considered significant. The data are presented as the mean ± standard deviation (SD). All analyses were performed using SAS 9.1 software.

## Results

### Plasma Viral Loads and CD4/CD8 Ratios in SIV-infected Animals

**Figure 1:**
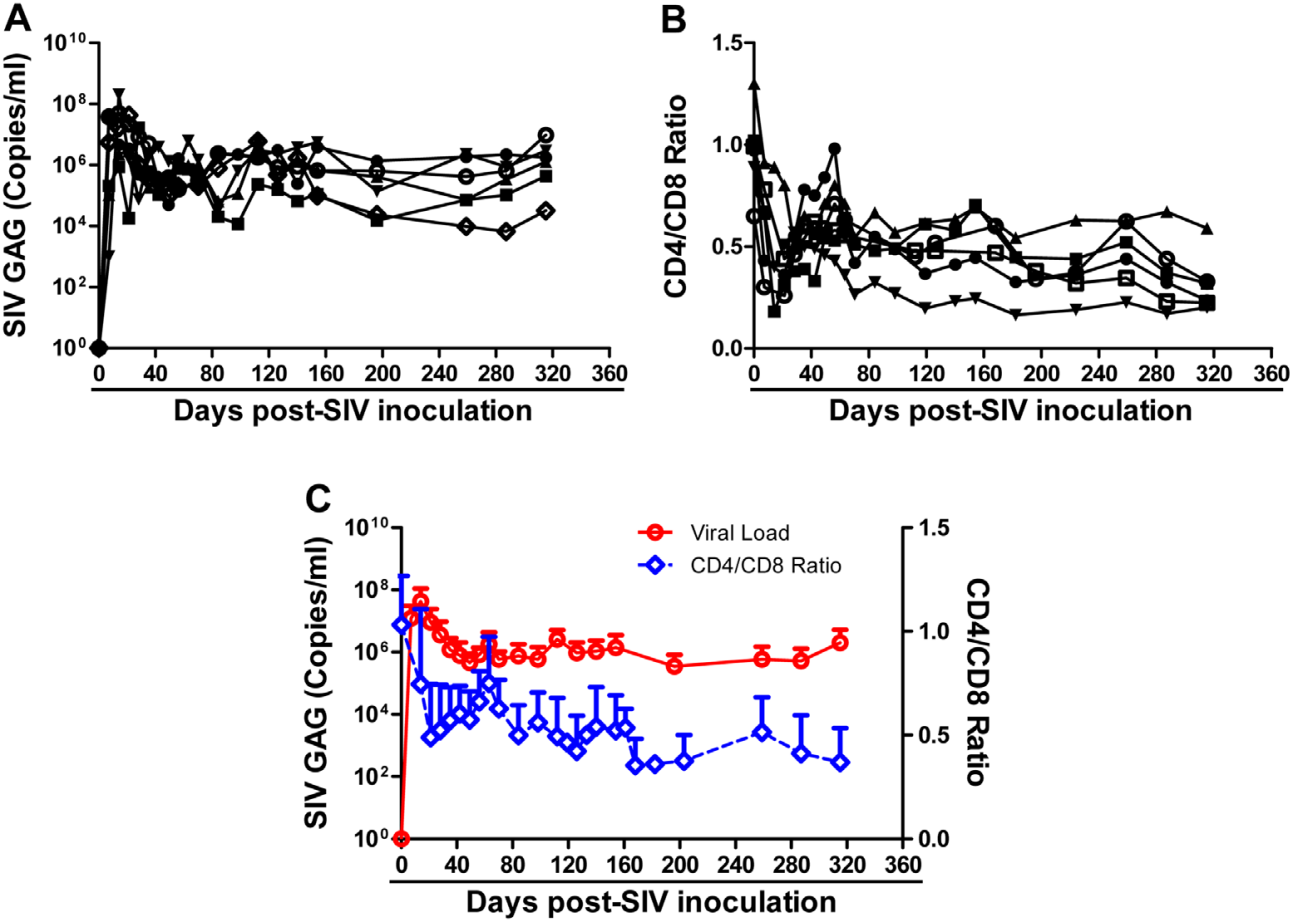
SIV infection of Chinese rhesus monkeys. Six animals were intravenously inoculated with SIVmac239 (10^3^ TCID50). Blood samples were collected from the animals at the indicated time points postinfection. A: Plasma levels of SIV GAG gene RNA were measured by real-time PCR. B: CD4/CD8 ratios were measured by flow cytometry. C: Average SIV loads and CD4/CD8 ratios (mean ± SEM) of SIV-infected animals.

Six animals were intravenously inoculated with SIVmac239 (10^3^ TCID50) and the viral loads in the plasma were monitored. All 6 animals became infected as evidenced by increased levels of plasma SIV gag RNA (Fig. 1A). The kinetics of plasma viral load were similar in all SIV-infected animals and the peak levels of the viral loads occurred at week 2 postinfection (Fig. 1A). A steady state of viral load reached at about day 80 postinfection (Fig. 1A). In the early time points postinfection, there was a significant decrease in the CD4/CD8 ratio in the infected animals (Fig. 1B), which subsequently recovered and became relatively stable at the lower levels than those prior to SIV infection (Fig. 1B). The changes of CD4/CD8 ratios were negatively associated with the viral loads during the course of SIV infection (Fig. 1C). At day 315 postinfection, all the infected animals remained to have CD4 cell numbers of over 350 cells/µL (ranging from 543 to1604) (Table 1).

### Effect of LPS Administration on Plasma SIV Load and Immune Activation

**Figure 2:**
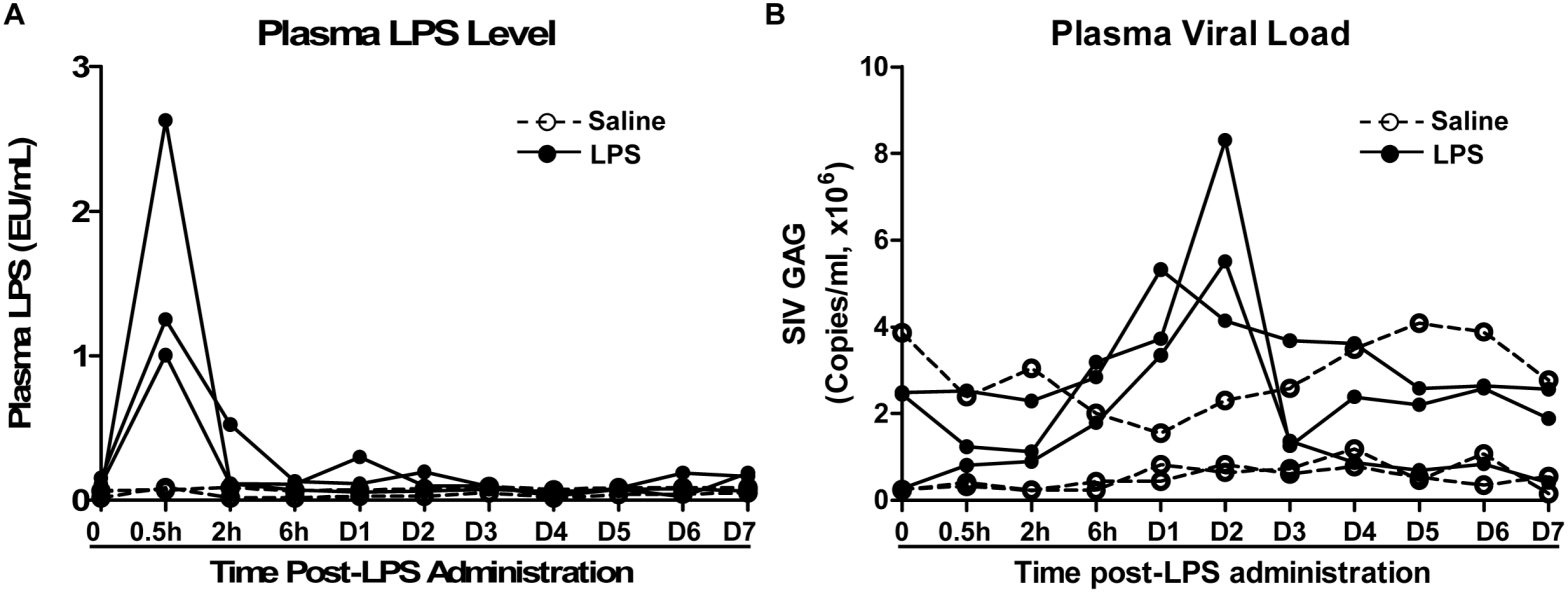
Effect of LPS on SIV replication. SIV-infected animals were intravenously injected with either a single dose of LPS (50 µg/kg; solid circles and lines, n = 3) or saline (open circle and dashed lines, n = 3) at 45 weeks postinfection. The plasma samples were collected at the indicated time points after LPS treatment. A: The plasma levels of LPS were determined by Tachypleus Amebocyte Lysate (TAL) test. B: SIV loads were measured by real-time PCR for SIV GAG gene expression.

**Figure 3:**
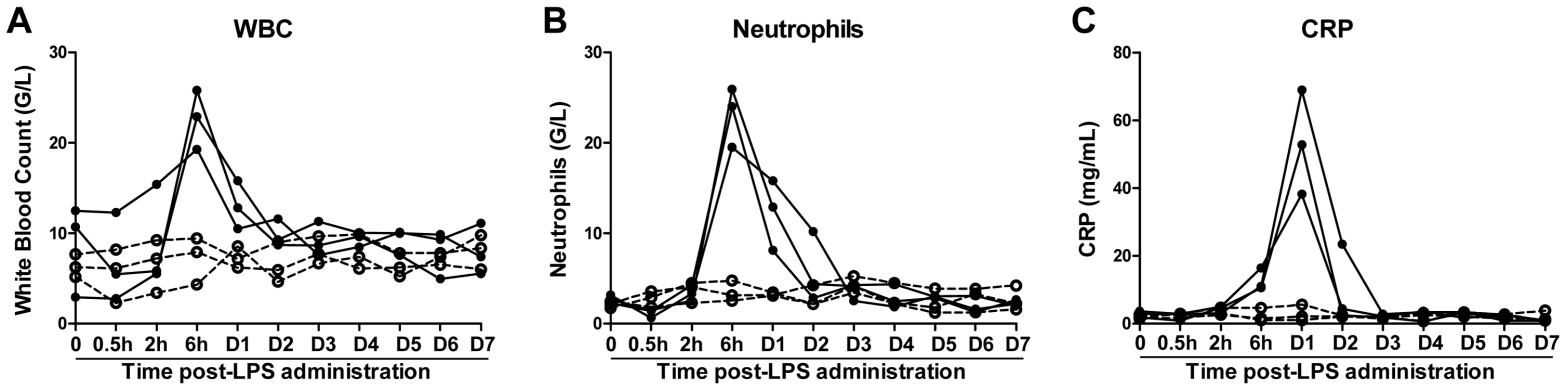
Effect of LPS on WBC, neutrophils and CRP. SIVmac239-infected animals were intravenously injected with either LPS (50 µg/kg; solid circles and lines, n = 3) or saline (open circle and dashed lines, n = 3) at 45 weeks postinfection. At the indicated time points after LPS administration, white blood cell (WBC) counts (A), neutrophil counts (B), and C Reactive Protein (CRP) levels (C) were determined by a biochemistry analyzer.

**Figure 4:**
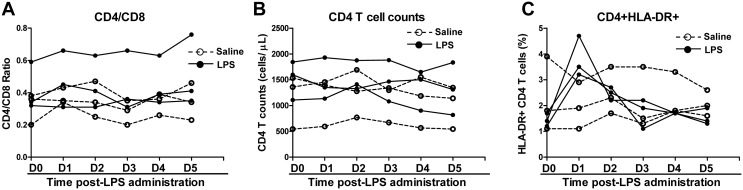
Effect of LPS on CD4/CD8 ratio, counts of CD4+ T cells and frequency of CD4+HLA-DR+ T cells. SIVmac239-infected animals were intravenously injected with either LPS (50 µg/kg; solid circles and lines, n = 3) or saline (open circle and dashed lines, n = 3) at 45 weeks postinfection. At the indicated time points after LPS administration, the CD4/CD8 ratios (A), total CD4+ T cell counts (B), and CD4+HLA-DR+ T cell percentage in PBMCs (C) were determined by flow cytometry.

We next examined the impact of LPS on SIV infection of Chinese rhesus macaques. The SIV-infected animals were administrated with LPS (n = 3) or saline (n = 3) at 45 weeks (315 days) postinfection. LPS levels in plasma could be detected after injection. As shown in Fig. 2A, the plasma LPS levels reached to a peak (1.0 EU/mL to 3.0 EU/mL) at 0.5 h post-administration and declined rapidly to the baseline at 2 h. LPS administration resulted in a rapid and transient increase of plasma SIV load, which peaked at 24 to 48 h post-treatment (Fig. 2B). In contrast, saline had little effect on the viral load in SIV-infected animals (Fig. 2B). LPS treatment of the animals induced the increase in the counts of WBC and neutrophils, both of which peaked at 6 h post-LPS administration (Fig. 3A, B). In addition, there was dramatic and transient increase in the expression of CRP, which peaked at 24 h post-LPS treatment (Fig. 3C). In contrast, saline had little effect on the counts of WBC and neutrophils as well as CRP levels of animals in the control group (Fig. 3). Although LPS had little effects on CD4/CD8 ratios and absolute CD4 T cell counts (Fig. 4A, B), LPS-treated animals had a transient increase in the frequency of CD4+ HLA-DR+ T cells as compared with the animals administrated with saline (Fig. 4C).

### Effect of LPS on Inflammatory Cytokines in PBMCs

**Figure 5:**
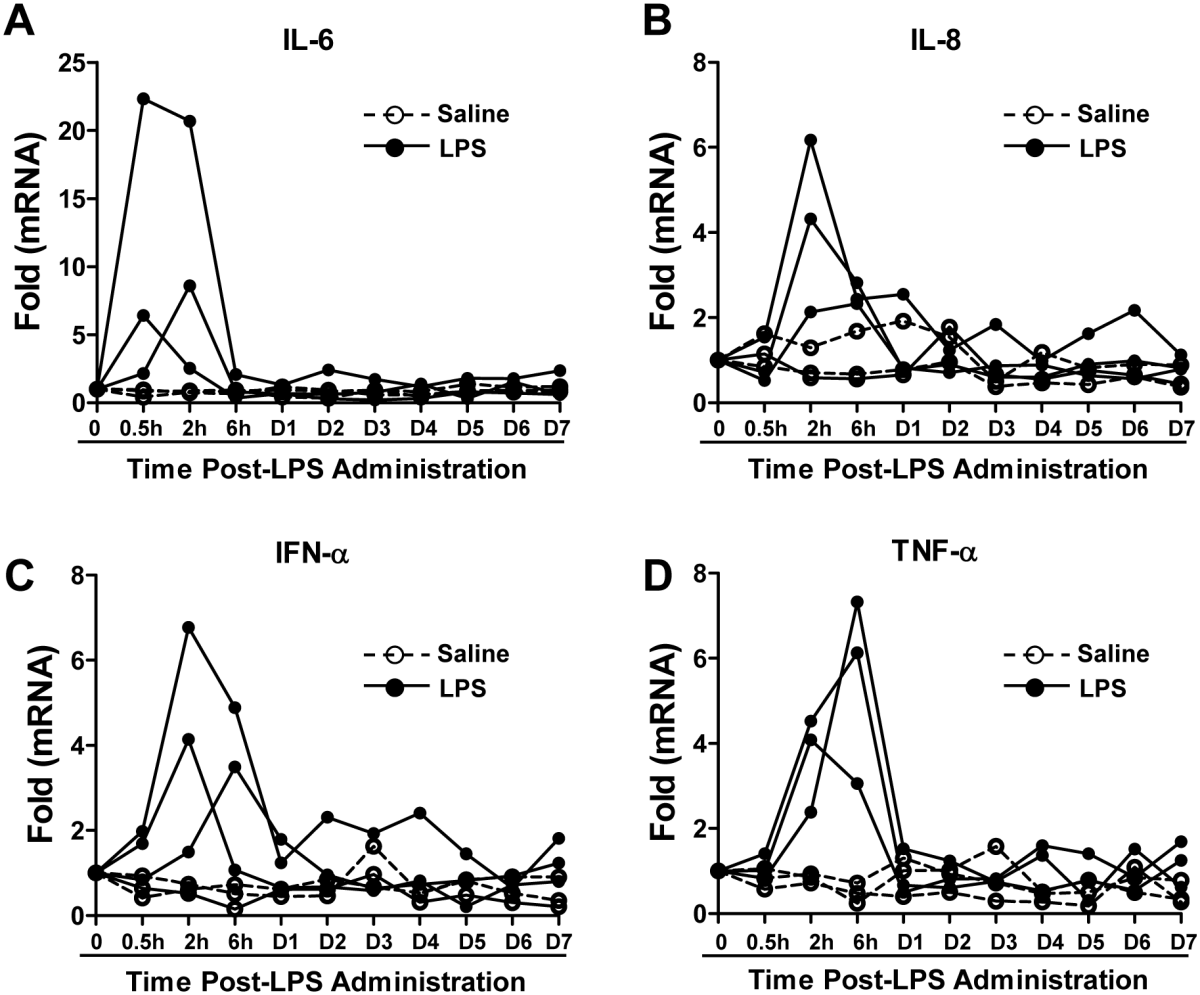
Effect of LPS on inflammatory cytokines in PBMCs. SIVmac239-infected animals were intravenously injected with a single dose of LPS (50 µg/kg, solid circles and lines, n = 3) or saline (open circle and dashed lines, n = 3) at 45 weeks postinfection. Blood samples were collected at indicated time points post-LPS administration and PBMCs were isolated. The levels of cytokines (IL-6, IL-8, IFN-α, and TNF-α) in PBMCs were determined by real-time PCR and normalized to GAPDH mRNA. Data are expressed as fold of control (before LPS administration, cytokine mRNA/GAPDH mRNA, which was defined as 1).

As LPS administration of infected animals enhanced SIV replication and induced CD4+ T cell activation, we next examined the impact of LPS on the expression of inflammatory cytokines in PBMCs. As shown in Fig. 5, compared to saline treatment, LPS administration induced a rapid and transient increase in IL-6, IL-8, IFN-α and TNF-α expression in PBMCs of SIV-infected macaques. This LPS-mediated induction of these cytokines peaked within 6 h post-LPS administration (Fig. 5).

## Discussion

Chronic immune activation is a common feature of progressive HIV disease. It has been shown that microbial translocation from the gut lumen into the systemic circulation is a cause of immune activation in either chronically HIV-infected patients [1], [15], [16] or SIV-infected rhesus macaques [17]. Translocation of bacterial bioproducts, such as LPS is a key facilitator of systemic immune activation [1]. Because of damage to GI tract by HIV infection, elevated plasma levels of LPS were found in chronically HIV-infected individuals [1], [18]. Elevated levels of plasma LPS were also observed in SIV-infected rhesus macaques [19]. However, we did not observe an increase of LPS in peripheral blood of SIV-infected Chinese rhesus macaques (data not shown). This discrepancy between our data and those from others could be due to the use of macaques of different origins (China origin v.s. Indian origin) and sensitivity of the assays used for LPS detection. To determine whether LPS is associated with systemic immune activation, we investigated whether LPS can experimentally induce immune activation in SIV-infected rhesus macaques of Chinese origin. We found that the bioactivity of administrated LPS was evidenced by the increased WBC counts, number of neutrophils and CRP in the circulation. The immune activation by LPS was demonstrated by the observation of increased expression of proinflammatory cytokines IL-6, IL-8, and TNF-α, a known factor involved in HIV activation [20], [21]. More importantly, LPS administration significantly induced T cell activation as measured by HLA-DR expression on CD4+ T cells (Fig. 4C). However, in contrast to the observations in the report by Pandrea et al [6], we did not observe the decrease of total number of peripheral CD4+ T cells. In addition, there was little change in CD4+/CD8+ T cell ratios in animals of the two groups. These discrepancies could be due to the different animal models (African green monkeys v.s. Chinese rhesus macaques) used in their and our studies.

Because HIV can replicate more efficiently in activated CD4+ T cells, chronic immune activation is responsible for providing more target cells for HIV infection, which contributes to the loss of CD4+ T cells. Similar to HIV infection, SIV infection of rhesus macaque could also result in a dramatic and selective loss of memory CD4+ T cells predominately from the mucosal surface in GI tract [22], [23]. This drastic depletion of CD4+ T cells in the intestine was also found in SIV-infected rhesus macaques of China origin [24], [25]. In contrast, SIV infection of natural host primates is associated with no microbial translocation and/or immune activation [17], [26]. These animals with or without SIV infection had similarly low levels of LPS [1]. However, experimentally induced immune activation in natural hosts of SIV could induce significant increases in viral replication and CD4^+^ T cell depletion [9]. To further determine direct causal relationship between LPS, immune activation and viral replication, we examined the impact of immune activation on SIV replication in Chinese rhesus macaques. We observed that LPS administration could transiently enhance SIV replication in Chinese rhesus macaques. This transient increase of peripheral blood SIV RNA was associated with plasma LPS levels, which rapidly declined to the baseline at 2 h post-administration (Fig. 2A).

Taken together, we have for the first time demonstrated that the administration of LPS could induce immune activation in rhesus macaques of Chinese origin, which was associated with immune activation and the increase of SIV replication in the infected animals. These findings provide direct and additional evidence to support the concept that microbial product LPS induces systemic immune activation and drives viral replication. The ongoing studies are necessary to investigate the immunopathogenesis of chronic SIV infection of Chinese rhesus macaques and the use of this model to determine whether the suppression of systemic immune activation can slow-down or prevent SIV- or SHIV-infected macaques from progression to AIDS. Given the detrimental impact of the microbial products (such as LPS) on overall host immunity, it is likely that to prevent/block LPS translocation and systemic immune activation is beneficial for people infected with HIV.

## Supporting Information

Checklist S1 **ARRIVE Guidelines.**
(DOC)
